# Deep Dysgraphia in Turkish

**DOI:** 10.1155/2005/568540

**Published:** 2006-01-09

**Authors:** Ilhan Raman, Brendan Stuart Weekes

**Affiliations:** ^1^Middlesex UniversityUK; ^2^University of SussexUK

**Keywords:** Deep dysgraphia, Turkish orthography, spelling deficits, phonological deficits, orthographic transparency, biscriptal writing

## Abstract

Deep dysgraphic patients make semantic errors when writing to dictation and they cannot write nonwords. Extant reports of deep dysgraphia come from languages with relatively opaque orthographies. Turkish is a transparent orthography because the bidirectional mappings between phonology and orthography are completely predictable. We report BRB, a biscriptal Turkish-English speaker who has acquired dysgraphia characterised by semantic errors as well as effects of grammatical class and imageability on writing in Turkish. Nonword spelling is abolished. A similar pattern of errors is observed in English. BRB is the first report of acquired dysgraphia in a truly transparent writing system. We argue that deep dysgraphia results from damage to the mappings that are common to both languages between word meanings and orthographic representations.

## 1. Introduction

Acquired dysgraphia is observed in many patients who have brain damage. Difficulties with writing can be classified into several different patterns [35]. These include surface dysgraphia which refers to poor spelling of irregularly spelled words, e.g. yacht, and a tendency to produce phonologically plausible spellings of irregular words, e.g. yot, accompanied by preserved spelling of regular words and nonwords [6,37,38]; phonological dysgraphia which is an inability to spell nonwords together with preserved spelling of words [4,7,33]; and deep dysgraphia which is an extreme form of phonological dysgraphia characterized by effects of lexicality, i.e. better spelling of words than nonwords, effects of imageability i.e. better spelling of highly imageable words such as elephant compared to less imageable words such as justice, and effects of grammatical class, i.e. better spelling of nouns than spelling of verbs in writing to dictation [7,16,31].

Subtypes of dysgraphia are distinguished by the ease with which a patient can predict the correct printed word output when given phonological input on tasks such as spelling to dictation. Most European languages contain ambiguity in the mappings between phonology and orthography and the evidence for dysgraphia subtypes has come from reports of patients who learned to read and write using orthographies with relatively opaque or irregular mappings between sound and print such as English and French [5,7,24,31]. Diagnoses of dysgraphia are thus linked to the predictability of mappings connecting phonology and orthographic output – specifically, the sound to spelling regularity of words. For this reason, the predictability of sound to spelling correspondences has been central to the development of cognitive models of spelling that are now widely used to diagnose impaired writing of patients who have acquired dysgraphia. The most influential of these is the dual-route model of spelling [13].

The dual-route model comprises two distinct routes for spelling to dictation: a nonlexical route for converting or assembling print from sound and a lexical route for retrieving or addressing previously stored spellings of familiar words. According to the model there are at least two inputs to the normal system used for written and oral spelling. The first is the semantic specification of the meaning of a word. This allows a proficient speller to spell correctly ambiguous words such

ISSN 0953-4180/05/$17.00 © 2005 – IOS Press and the authors. All rights reserved as homophones e.g., SAIL-SALE. The second input is a phonemic specification of the sound of the word to be written. This input comes from the phonological word system or the output lexicon used for normal spoken word production. Input from this system explains why normal slips of the pen are sometimes characterised by phonological similarity to the target e.g. 'surge' for SEARCH. In the extended three-route model [14], the lexical route can be further subdivided into a direct-lexical and a lexical-semantic route. Nonwords and words with regular sound to print mappings are assumed to be spelled using the nonlexical route whereas words with irregular sound to print mappings are assumed to be spelled using the lexical route thus preventing incorrect production of phonological spelling errors, at least in less transparent orthographies. Phonological dysgraphia has been interpreted within the dual-route model as impaired assembly of print from sound via the nonlexical route (hence nonword spelling is poor) whilst previously learned, familiar words are spelled via the lexical route which is intact. Surface dysgraphia reflects impaired spelling via the lexical route accompanied by a preserved nonlexical route leading to poor spelling of irregularly spelled words that are regularised spellings of the target e.g., *yot* (see [15] for a slightly different view). Deep dysgraphia results from severe damage to the spelling system. Exclusive use of the lexical-semantic route is assumed to explain why effects of lexicality, imageability and grammaticality as well as semantic errors are seen in deep dysgraphia [7,16,27].

One important question about acquired dysgraphia is the role of orthographic transparency across different scripts in the diagnosis of spelling errors. Of particular interest are languages with predictable mappings between phonology and orthography such as Italian and Spanish. Some writers argue that acquired dysgraphia should not exist in transparent orthographies [2]. This is because correct spelling can be achieved a) using a nonlexical route so that lexical knowledge is redundant for spelling and b) equally successfully by using either the nonlexical or the lexical route so that damage to one will not result in a disorder of spelling. However there are reports of acquired surface dysgraphia in Spanish [20]. The lexical route assumed in the dual-route model may therefore be available for normal spelling in relatively transparent orthographies and thus may be selectively impaired in patients with aphasia [36] (for similar arguments about reading in Spanish see the papers by Davies and Cuetos and by Ferreres this volume].

Reports of developmental dysgraphia [1] and acquired dysgraphia [22,26] in Italian further support this view and suggest that lexical knowledge may be necessary to spell some words in transparent orthographies. Cappa et al. [8] reported deep dysgraphia in Italian characterised by semantic errors (paragraphias) and effects of grammatical class (nouns better than verbs) and imageability on writing to dictation (but not reading aloud or repetition) as well as complete inability to write nonwords. According to the dual-route model, deep dysgraphia occurs if nonlexical knowledge used to spell nonwords is abolished, revealing the operation of the lexical-semantic spelling route. The finding that deep dysgraphia can be observed in Italian thus supports the idea that both direct-lexical and lexical-semantic routes are used to spell in transparent orthographies.

The transparency of sound to spelling mappings in Italian and Spanish are overstated in our view since sound to spelling irregularities exist in both orthographies [3,9,32]. Therefore, it is perhaps not surprising that lexical knowledge plays a role in spelling Italian and Spanish words. It is of greater interest to examine impairments to spelling in Turkish which is a writing system that is completely transparent so that orthographic output can be predicted from sound without any lexical knowledge.

## 2. Turkish orthography

The modern Turkish orthography comprises an adapted version of Latin with 29 letters; eight vowels and 21 consonants. The most salient feature of Turkish orthography is the one-to-one mappings between orthography and phonology. The orthography was deliberately designed to eliminate sources of irregularity from the script particularly where words that were Turkish in origin are concerned.[1] The alphabet represents the sounds in the spoken language in a transparent manner, where both orthography to phonology (reading) and phonology to orthography (spelling to dictation) translations are regular, explicit and predictable. That is, pronunciation of words and nonwords in reading aloud, i.e. mappings from orthography to phonol-

[1] It should be noted that as in other languages there are loan words in Turkish which may not necessarily fit into the orthography to phonology mapping profile described above. This is particularly true for a minority of loan words that are Arabic in origin with long vowels. However, we did not use these words in our testing. We thank an anonymous reviewer for making this point.

ogy, and spelling words and nonwords to dictation, i.e. mappings from phonology to orthography, is correctly predicted in the absence of lexical information. This is because each letter in the orthography always maps onto the same sound. Vowel harmony is another important property of Turkish in that morphological inflections are generated according to two discrete categories of vowels, namely front (E, İ, Ö, Ü) and back vowels (A, I, O, U). To give an example, two forms of the plural suffix exist: -LER is used in words where the front vowels E, İ, Ö, Ü occur, as in üzüm-ler *grape-s*, whilst -LAR is used in the presence of back vowels A, I, O, U, e.g. kutu-lar *box-es*. For foreign loan words, such as televizyon-lar *television-s*, vowel harmony is maintained according to the last syllable (-YON) which contains the back vowel $o$. Similarly, risk-ler *risk-s* takes the plural suffix -LER in accordance with the front vowel $i$, again preserving vowel harmony. Thus Turkish contains invariant and context independent mappings between orthography and phonology as well as a high number of vowels. This means that the Turkish orthography is a more transparent script than Italian and Spanish, at least in the mappings between phonology and orthography.

We report patient BRB who has acquired dysgraphia in Turkish and English. BRB's pattern of spelling errors conforms to the typical features of deep dysgraphia in relatively opaque languages, suggesting that a lexical-semantic spelling process is available for spelling in Turkish. This is the first report of acquired deep dysgraphia in a completely transparent writing system.

## 3. Case report

BRB is a right-handed 67 year old man who had a cerebrovascular accident (CVA) in November 1999. BRB suffered severe loss of speech after the CVA and a CT scan revealed a medium sized left temporal parietal-occipital lobe infarction (shown in Fig. 1). BRB is a retired senior civil servant from Cyprus where his native language was Turkish, which was a co-official language with English and Greek (Cyprus was a British colony until 1960). BRB was educated in English between the ages of 11–21 and he used both languages daily in his adult life. BRB's premorbid IQ was estimated in the average range based on previous education and work history.

BRB's performance on tasks conducted in Turkish and English including oral reading, spoken naming of pictures, repetition, written and spoken picture-word matching is reported in full elsewhere [29,30]. In brief, BRB was able to complete written and spoken word-picture matching tests (making only two errors which was no worse than controls) showing he recognised objects and understood the meaning of printed and spoken words. BRB is anomic and has problems retrieving phonological output on picture naming tasks in Turkish and English. He produced semantic errors e.g., thumb *başparmak* → finger *parmak* although phonological errors were also observed e.g., rabbit – raffit. His repetition was poor and abolished for nonwords consistent with a diagnosis of deep dysphasia [29]. BRB's poor repetition of words and nonwords suggests impairment to phonological representations in both languages as well as impairment to mappings between semantics and phonology/orthography. BRB wrote with his dominant right hand, and his writing was perfectly legible. There was no evidence of any noticeable change to his calligraphy following the stroke.

## 4. Experimental investigations

Our first hypothesis was that because of BRB's nonword repetition disorder, he would be deep dysgraphic. This was based on previous reports that patients with repetition disorders produce deep dygraphic spelling errors in French [23] and Italian [8]. Also, we had previously identified an effect of imageability on BRB's reading in Turkish and an effect of regularity on his reading in English suggesting he used a lexical-semantic route to read in both languages [30]. We used the methods developed by Cappa et al. [8] who identified deep dysgraphic symptoms in Italian. They defined deep dysgraphia in terms of a) inability to write nonwords b) semantic errors in writing and c) significant effects of grammatical or word class (nouns spelled better than verbs) and effects of imageability on writing. We examined writing performance in both languages using the same items (where possible) to assess the effects of script transparency on written word output (English is less transparent than Turkish).

Task a) Spelling nonwords

BRB was presented with 40 nonwords to spell to dictation. It is not possible to distinguish nonwords in terms of language (Turkish or English). However, BRB was given verbal instructions in Turkish for the task and asked to spell nonwords from dictation using the rules of Turkish (stimuli are reported in the Appendix). He was also told that some stimuli may not be real words

Table 1
Number and percentage correct on tests of spelling to dictation using Turkish nouns, verbs and derived nouns for BRB

| Nouns | | Verbs | | Derived verbal nouns | | Nonwords | |
|---|---|---|---|---|---|---|---|
| Correct 22/34 | (64%) | Correct 3/34 | (9%) | Correct 0/34 | (0%) | Correct 0/40 | (0%) |
| Type of error | | Type of error | | Type of error | | Type of error | |
| No response | 9 | No response | 8 | No response | 6 | Unrelated 40/40 | |
| Semantic | 3 | Semantic | 0 | Semantic | 0 | | |
| Nonword | 0 | Nonword | 15 | Nonword | 14 | | |
| | | Unrelated | 8 | Unrelated | 9 | | |
| | | | | Related | 5 | | |

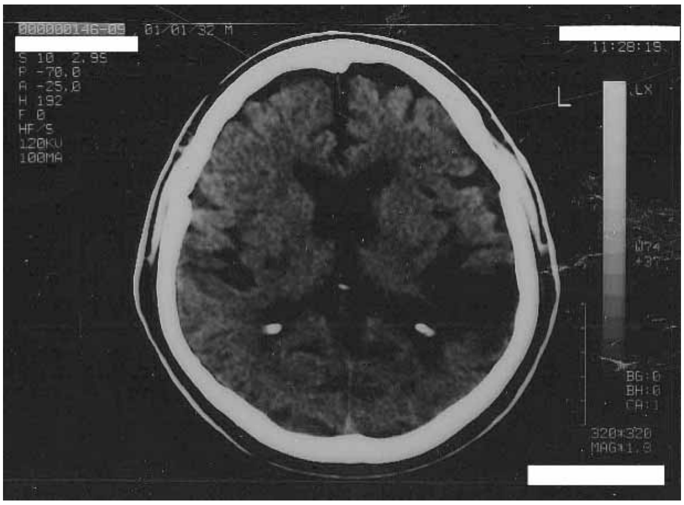

Fig. 1. CT scan showing BRB's left temporal lesion after CVA.

but he should nevertheless try and spell them as if they were real words in Turkish. BRB could not spell any of these stimuli, scoring 0/40 correct.

Task b) Spelling nouns, verbs and derived verbal nouns in Turkish

The next test examined noun and verb processing, i.e. effects of word class and imageability on writing to dictation. BRB was given 34 concrete nouns (ağaç *tree*); 34 verbs in the infinitive (açmak *to open*); and 34 derived verbal nouns (gülme *the act of laughing*) to spell to dictation in Turkish (see Appendix for stimuli). The 34 derived verbal nouns were generated from verb stimuli by using the morphological properties of Turkish. Note that some verbal nouns (13/34) were also polysemous, e.g. asma *the act of hanging/vine tree*. Results are presented in Table 1.

BRB's performance on writing to dictation was 64% correct for nouns, 9% correct for verbs and 0% correct for derived nouns. He produced semantic errors in writing as exemplified by the following; AĞAÇ *tree* written as BAHÇE *garden*; AYAKKABI *shoe* as KUNDURA *local flat shoe* and GÜNEŞ *sun* as GÜNDÜZ *day*. There was a significant effect of word class on spelling $F(2, 66) = 47.82$, $p < 0.01$. Analyses of simple main effects ($p < 0.05$) showed that he spelled nouns significantly better than verbs, which were in turn spelled better than verbal nouns. Nouns and verbs are difficult to match on lexical variables such as however imageability, age of acquisition and length [11]. Items in the Appendix were not matched for imageability prior to testing. However imageability ratings were subsequently obtained from twenty native Turkish speakers (undergraduate and postgraduate students from North Cyprus) using a 7-point Likert scale (high imageability = 1 and low imageability = 7 on this scale). The descriptive statistics on this variable were for nouns (mean = 1.64, sd = 0.30); verbs (mean = 3.94, sd = 0.37) and derived verbal nouns (mean = 3.95, sd = 0.32). Verbs and verbal nouns did not differ on rated imageability $t(66) = 0.13$, $p = 0.9$. However nouns were significantly more imageable than verbs $t(66) = 28.31$, $p < 0.0001$ and verbal derived nouns $t(66) = 31.13$, $p < 0.0001$. These results show that the effect of word class on BRB's spelling could be due to differences in rated imageability rather than grammatical class. Data were analysed further by assessing effects of imageability and word class using regression analysis. The overall regression equation was significant, $F(2{,}99) = 44.42$, $p < 0.001$, $R = 0.688$, where the two factors account for 46% of the variance. However, imageability was the only significant predictor of spelling errors and the effect of word class was not significant. Therefore the dissociation between spelling nouns, verbs and derived nouns is most likely due to the influence of imageability. An imageability effect is a hallmark of deep dysgraphia in other languages and suggests that BRB was spelling words using a lexical-semantic route, albeit one that is not functioning optimally.

Task c) Spelling in English

The results above suggest that BRB is deep dysgraphic and following Cappa et al. [8] that he uses a lexical-semantic route to spell Turkish words. It was of interest to assess whether he uses a lexical-semantic route to spell in English too since this would provide a test of our claim that orthographic transparency might moderate spelling impairments. Our intuition was that if BRB spells using a damaged lexical-semantic route, then homophone spelling in English ought to be impaired since this ability uses contextual knowledge i.e., the meaning of the homophone will determine how it is spelled. This hypothesis can only be tested in English, as there are no homophones in Turkish. BRB was asked to write to dictation twenty homophonic words e.g. *pain* taken from the Psycholinguistic Assessment of Language Processing and Aphasia (PALPA) battery [21]. Stimuli were presented in context e.g., The *pain* in his leg hurts, by giving him written sentences followed by a space to write the target words, and the full sentence including the word to be written was dictated by the experimenter 'The _____ in his leg hurts' with the verbal instruction, 'Write the word pain'. Results are in Table 2.

BRB scored 11/20 correct (55%) and he made semantic errors in spelling e.g. the target word pain was substituted with the semantically related but incorrect word *wound*. Thus, as expected he was impaired at homophone spelling that must depend on a lexical-semantic route [13,14]. BRB has impairment to the lexical-semantic route when spelling in both languages. The evidence suggests that spelling errors in English and in Turkish result from abolition of the nonlexical route for spelling accompanied by the use of a lexical-semantic route that is damaged. But where is the lexical-semantic route damaged?

Task d) Writing picture names in English and Turkish

One possible locus of damage to the lexical-semantic spelling route is in the mappings between semantics and orthography. We can assume that writing picture names in English involves accessing orthography from semantics directly and does not require phonological mediation [34]. Thus we expected BRB to be impaired when writing picture names in English. However, if lexical knowledge is redundant when writing picture names in a transparent script (as some might argue) then performance in Turkish could be intact. BRB was given 40 items from PALPA Test 53 and asked to write picture names directly from pictures in English and Turkish and to spell the same names from dictation. His results are summarised in Table 3. Performance was comparable in both languages. BRB was impaired on written picture naming, 82% correct in English and 77% correct in Turkish; and spelling to dictation, 77% correct in both languages. Of interest is the similarity of spelling errors that BRB produced when writing words in English and Turkish (see examples in Table 3).

## 5. Summary

The data suggest that BRB's access to orthographic output from the semantic system is impaired (as is access to phonological output resulting in anomia) and this has an equivalent effect on spelling in Turkish and English. The data thus point to the lexical-semantic route as the likely locus of damage leading to deep dysgraphia for BRB. Our original hypothesis was that writing and spelling would be impaired for BRB because of repetition problems and damage to the phonological representations that must be used to spell to dictation. However, we found that written picture naming (which does not require phonological mediation) is also impaired. Thus, although BRB has problems when spelling from phonological input (impaired spelling to dictation) and spelling via the nonlexical route (as nonword spelling is abolished), there is additional impairment in the mappings that link semantics to orthography in both languages. This pattern can be explained by the dual-route model as it assumes a lexical-semantic route to orthographic output without phonological mediation. The present data complement that model by showing that a lexical-semantic route – albeit damaged – can be used to spell in Turkish.

Table 2
Number and percentage correct on tests of homophone spelling in English for BRB

| Sentences in English | Target | Response/ Nature of error |
|---|---|---|
| He walked the *Great* Wall of China. | great | great |
| She went *pale* with shock. | pale | pale |
| You must have a tea *break*. | break | **bread PHON*** |
| The *pain* in his leg hurts. | pain | **wound SEM** |
| Mum is kneading the *dough* to make bread. | dough | **slice SEM** |
| He took a *loan* to pay for his car. | loan | loan |
| The regiment took their orders from their *colonel*. | colonel | **colnel PHON** |
| The postman delivered the *mail*. | mail | mail |
| The polar *bear* is white. | bear | bear |
| The saucepans are made of stainless *steel*. | steel | steel |
| My cat likes to *roll* on her back. | roll | **no response** |
| The horses *haul* the cart. | haul | **ride SEM** |
| Charles is the *heir* to the throne. | heir | heir |
| *Meat* is a good source of protein. | meat | meat |
| The business man stayed at the most expensive hotel *suite*. | suite | suite |
| Mohammed was the last *prophet*. | prophet | prophet |
| The swimming-pool had a water *chute*. | chute | **no response** |
| I am going away for a *week*. | week | week |
| She always makes a *scene*. | scene | **run UR** |
| He walks with an unsteady *gait*. | gait | **stagger SEM** |
| Correct | | 11/20 (55%) |
| Type of error | | |
| Semantic | | 4/9 (44%) |
| No response | | 2/9 (22%) |
| Phonological | | 2/9 (22%) |
| Unrelated | | 1/9 (11%) |

*Note that errors are in bold and categorised as follows: SEM denotes a semantic error; NR a no response; NW a nonword; UR a word unrelated to target; PHON a phonological error.

## 6. Discussion

The data from BRB reveal a number of facts about spelling and writing in a transparent orthography. First, these are the first data showing acquired dysgraphia in Turkish, the prime example of a script with completely predictable mappings between orthography and phonology. Therefore, the naive view that acquired dysgraphia will not be observed in transparent orthographies can be rejected. Second, these data alert the clinician to the possibility of deep dysgraphia in Turkish which is comparable to the findings of Cappa et al. [8] who reported deep dysgraphia in Italian, another highly transparent orthography (see also Davies and Cuetos this volume).

The potency of the present data is that deep dysgraphia is observed in a language with completely predictable mappings between phonology and orthography. Also, the similarity in the pattern of spelling errors observed in English and Turkish suggests that biscriptal spelling depends on common cognitive mechanisms, at least in two alphabetic languages that are highly practiced in this patient. Finally, if our assertions are correct then lexical knowledge is clearly not redundant for spelling in transparent scripts and spelling in Turkish can be achieved using a lexical-semantic route.

Why does BRB make semantic errors when spelling to dictation? Miceli et al. [25] offered an account of semantic errors in Italian that was based on the summation hypothesis first proposed by Hillis and Caramazza [17,18]. Within that model of lexical-semantic processing, the consequences of impairment to a nonlexical spelling mechanism will differ according to the transparency of the script. One assumption in Miceli et al.'s [25] account is that semantic errors in transparent orthographies will only be observed following complete abolition of nonlexical conversion procedures. This is because assuming an interaction between outputs from the lexical-semantic and nonlexical routes on activation in the orthographic output lexicon as specified by Hillis and Caramazza [17,18], then even a partial sparing of nonlexical mechanisms would be sufficient to block the production of semantic errors in transparent scripts. However, semantic errors may be observed in languages that contain irregular sound to spelling mappings following only partial damage to a

Table 3
Number and percentage correct from PALPA Test No. 53 in English and Turkish for BRB

| Picture names – Turkish translation | Written picture naming in English | Spelling picture names to dictation in English | Written picture names in Turkish | Spelling picture names to dictation in Turkish |
|---|---|---|---|---|
| comb *tarak* | comb | comb | tarak | tarak |
| bear *ayı* | bear | **buesh NW*** | ayı | ayı |
| horse *at* | horse | horse | at | at |
| mountain *dağ* | mountain | mountain | dağ | dağ |
| screw *vida* | screw | **no response** | vida | vida |
| anchor *demir* | anchor | anchor | demir | demir |
| glove *eldiven* | glove | glove | **eldevirir NW** | **mevdiren NW** |
| belt *kolan/kayış* | belt | belt | kolan | kayış |
| cow *inek* | cow | cow | inek | inek |
| fork *çatal* | fork | fork | çatal | **iskemele NW** |
| arrow *ok* | arrow | **no response** | ok | ok |
| bowl *çanak* | **vase SEM** | bowl | **voza NW** | **no response** |
| chair *sandalye* | chair | chair | sandalye iskemle | **no response** |
| glass *bardak* | glass | **no response** | bardak kadeh | bardak |
| bread *ekmek* | bread | bread | ekmek | ekmek |
| shoe *ayakkabı* | shoe | shoe | **kundura SEM** | ayakkabı |
| iron *ütü* | iron | iron | ütü | ütü |
| elephant *fil* | elephant | elephant | fil | fil |
| swan *kuğu* | swan | swan | kuğu | **köpek SEM** |
| heart *kalp* | heart | **no response** | kalp | kalp |
| eye *göz* | eye | eye | göz | **no response** |
| bird *kuş* | bird | bird | kuş | kuş |
| monkey *maymun* | **animal SEM** | monkey | maymun | maymun |
| ladder *merdiven* | **stair SEM** | ladder | **mendir NW** | merdiven |
| rabbit *tavşan* | rabbit | rabbit | **taşvan NW** | tavşan |
| star *yıldız* | star | **no response** | yıldız | yıldız |
| brush *fırça* | brush | **no response** | fırça | **no response** |
| thumb *başparmak* | **finger SEM** | thumb | başparmak | başparmak |
| scissors *makas* | **scirres NW** | scissors | makas | makas |
| toaster *ızgara* | **toast SEM** | **bread SEM** | **toast SEM** | ızgara |
| watch *saat* | watch | watch | saat | saat |
| seal *fog balığı* | seal | seal | **ayı balık SEM** | fog balığı |
| dog *köpek* | dog | dog | köpek | köpek |
| yacht *yat* | **sail SEM** | yacht | **yat yelken SEM** | yat |
| foot *ayak* | foot | foot | ayak | **dağ UR** |
| swing *salıncak* | swing | swing | **masa UR** | salıncak |
| lemon *limon/ekşi* | lemon | lemon | **lemon ENG** | limon |
| knife *bıçak* | knife | knife | bıçak | bıçak |
| fish *balık* | fish | **tooster NW** | balık | **fog balık SEM** |
| onion *soğan* | onion | onion | soğan | soğan |
| | Correct 33/40 (82%) | 31/40 (77%) | 30/40 (77%) | 31/40 (77%) |
| | Type of error | Type of error | Type of error | Type of error |
| | Semantic 6/7 | Semantic 1/9 | Semantic 4/9 | Semantic 2/9 |
| | No response 0 | No response 6/9 | No response 0 | No response 4/9 |
| | Nonword 1/7 | Nonword 2/9 | Nonword 4/9 | Nonword 2/9 |
| | Unrelated 0 | Unrelated 0 | Unrelated 1/9 | Unrelated 1/9 |

nonlexical processing route. The reasoning for this is that the division of labour for spelling in less transparent scripts emphasises the use of the lexical-semantic route. Our data support Miceli et al.'s [25] account as the nonlexical route for spelling is clearly not available for BRB and as predicted semantic errors in writing are produced.

We attribute BRB's spelling deficits in Turkish and English to the abolition of a nonlexical spelling route and a partially damaged lexical-semantic route. The locus of damage to the lexical-semantic route is in the mappings between semantic memory and orthographic output given BRB's performance on tests of picture name writing. Production of semantic errors and significant effects of rated imageability on spelling also suggest BRB uses a lexical-semantic route to spell – albeit imperfectly. Writing picture names in both languages also shows there is damage to mappings between the lexical-semantic representations and orthography. This is not a surprising outcome when considering spelling in English because the inconsistent mappings between orthography and phonology means that additional input from semantic memory can prevent errors in normal spelling. Moreover, if these mappings are damaged then a pattern of deep dysgraphia should result. It is more surprising that damage to the lexical-semantic route has an impact on spelling in Turkish.

Despite the phonological deficits that are evident in BRB's difficulties when converting sound to print, another surprising observation was that BRB rarely violated vowel harmony when he generated nonword substitutes for verbs and derived nouns baring two exceptions (icarmak and mekatmek). By contrast he violated the same rule for nonwords almost all of the time. We take this to mean that vowel harmony in Turkish is a categorical constraint, which may be orthogonal to phonological deficits.

One interesting observation was that BRB reverted back to spelling the verb in the infinitive from derived nouns. Turkish is an agglutinating language in which words co-exist in root and inflected form. It is not yet known whether inflected words in Turkish or in other highly inflected languages have distinct representations in the language system. Our proposal here is that there is a constraint on the cognitive system in terms of organising and bringing together multi-inflections and that this is a characteristic of Turkish. Durgunoglu [12] suggests that morphological inflections for agglutinated words are assembled such that representations for inflected words cannot exist as single entities. Derived nouns in this respect are morphologically and semantically complex when compared to nouns, which presumably correspond to unique entries in semantic memory. The discrepancy between categorical overlap between word classes could be magnified by a partially functioning lexical-semantic route such that the target class type, i.e. the verb in the infinitive, is preferred to the derived form. Insofar as derived nouns and nouns proper are concerned, the pattern of results here points to a possible dissociation between the two word categories. Processing derived nouns in Turkish appears to make disproportionate demands on the semantic system. BRB is relatively proficient when spelling simple nouns, but not verbs or derived verbal nouns. We assume that highly inflected Turkish words are not stored as distinct representations, but a computational system is used to assemble words [12]. To give an example, çizmek *to draw* is the infinitive form of the verb çiz *draw*, but when it is in its derived form, it can be a) a completely unrelated noun çizme *boot*; b) a related noun çizme *act of drawing*; and c) çizme *command: don't draw*. Each of these derived words varies in the type of further suffixes they can take, e.g., çizmeler *boots*; and çizmede *during the act of drawing/in the boot*. With the above in mind, we further speculate that BRB's difficulty when spelling verbs and verbal nouns would be further exaggerated in spelling tasks demanding the generation of such morphologically complex variant suffix alternatives. Nouns were all in their simple root form in the present study. If our morphology account is correct, we would also expect to observe difficulties in the inflection of nouns, e.g., bahçelerde *in the gardens* as opposed to bahçe *garden*, with other patients who have a similar profile to BRB.

As a biscriptal reader BRB gave us the opportunity to explore how damage to the spelling system manifests itself according to orthographic transparency. BRB has mostly equivalent difficulty spelling words in English and Turkish and impairment to retrieval of orthographic output for words in both languages. On the whole, the data show that damage to the mappings that support phonology to orthography conversion will have a consequence for both languages of a biscriptal reader although these may manifest differently according to the properties of the script and the type of task.

BRB's deep dysgraphia results from over-reliance on a compromised lexical- semantic route. Our assertion is compatible with other patient data [19]. Specifically, impairment at the level of the lexical-semantic spelling route affects reading of irregular words and low imageability words in both scripts. BRB's spelling to dictation was compromised and there was an effect of word imageability on spelling in Turkish i.e. high imageable nouns were spelled better than less imageable verbs which were in turn spelled better than verbal nouns. This suggests that relatively proficient Turkish readers may need to apply more cognitive effort when spelling words that are low in imageability and/or morphologically complex. A further assertion is that phonological impairments i.e. poor repetition and problems with writing to dictation are linked in Turkish. This has some implications for the assessment of developmental dysgraphia.

Tainturier and Rapp [34] argued that selective deficits to nonword spelling and writing to dictation in languages with transparent orthographies strengthen theoretical claims about distinct lexical and nonlexical spelling procedures. This is because there is a less obvious need for a lexical spelling process in transparent orthographies when compared to the more intensively studied alphabetic languages such as English and French. The present study show that it is possible to spell all words in Turkish using nonlexical sound to spelling mappings given contextual knowledge is not necessary to spell. However, there is a secondary lexical-semantic system that can be used to spell in Turkish when the nonlexical system is unavailable and this is revealed in patients with deep dysgraphia. The data from BRB should encourage clinicians to examine patients for signs of deep dysgraphia in Turkish and highlight the similar architecture of the spelling system in different languages.

The two main conclusions can be drawn from BRB's results: first that lexical- semantic knowledge plays a role in the spelling of Turkish words and second the effect of orthographic transparency on the translation of phonology to orthography is minimal at least in a proficient biscriptal Turkish-English speaker. What seems most critical to explaining the pattern of acquired dysgraphia across scripts is that damage to the lexical-semantic and nonlexical mechanisms in spelling will produce a variety of dysgraphias irrespective of the unique properties of the orthography. This implies that normal writing (and reading) in Turkish uses input from the semantic system (see [28,29] for a discussion) and that more than one route is available for spelling (and reading) in Turkish as is in other transparent scripts [34].

## Acknowledgements

We thank Judit Druks and an anonymous reviewer for valuable comments on a previous draft of the paper.

## Appendix

Turkish nouns, verbs and derived nouns with English translations. Polysemy is indicated by highlighting the additional meaning in bold.

| Noun Translation | Verb Translation | Derived Verbal Noun Translation **Noun** |
|---|---|---|
| asma *the act of hanging*/**vine** | | |
| aday *candidate* | asmak *to hang* | açma *the act of opening*/**type of bread** |
| anne *mother* | açmak *to open* | akma *the act of flowing* |
| ağaç *tree* | akmak *to flow* | atma *the act of throwing* |
| ateş *fire* | atmak *to throw* | aldatma *the act of adultery* |
| ayakkabı *shoe* | aldatmak *to commit adultery/to cheat* | anma *the act of remembering* |
| ayna *mirror* | anmak *to rememeber* | biçme *the act of reaping* |
| bahçe *garden* | biçmek *to reap* | bölme *the act of dividing*/**math. division** |
| balık *fish* | bölmek *to divide* | basma *the act of stepping/printing*/**printed cotton** |
| balkon *balcony* | basmak *to step/to print* | bilme *the act of knowing* |
| bardak *glass* | bilmek *to know* | boğma *the act of drowning* |
| bebek *baby/doll* | boğmak *to drown* | çarpma *the act of colliding with*/**math. multiplication** |
| çocuk *child* | çarpmak *to strike/collide with/multiply* | çizme *the act of drawing*/**boot** |
| çiçek *flower* | çizmek *to draw* | dövme *the act of beating*/**tatoo** |
| defter | dövmek *to beat* | dolma *the act of filling*/**stuffed leaves** (**food**) |
| excercise *book* | dolmak *to fill* | delme *the act of piercing* |
| deniz *sea* | delmek *to pierce* | doğma *the act of being born* |
| doktor *doctor* | doğmak *to be born* | ezme *the act of crashing*/**mashed dish** (**food**) |
| dünya *earth/world* | ezmek *to crash* | eşme *the act of scratching the surface* |
| ekmek *bread* | eşmek *to scratch the surface* | geçme *the act of passing* |
| erkek *male/man* | geçmek *to pass* | gezme *the act of strolling*/**stroll** |
| gazete *newspaper* | gezmek *to stroll* | gitme *the act of going* |
| gece *night* | gitmek *to go* | gülme *the act of laughing* |
| giysi *clothes* | gülmek *to laugh* | haşlama *the act of boiling* |
| güneş *sun* | haşlamak *to boil* | inme *the act of descending*/**stroke** |
| haber *news* | inmek *to decend* | kırma *the act of breaking*/**pleat** |
| insan *human* | kırmak *to break* | kazma *the act of digging*/**pickaxe** |
| kitap *book* | kazmak *to dig* | olma *the act of being* |
| kardeş *brother* | olmak *to be* | parlama *the act of shining* |
| okul *school* | parlamak *to shine* | satma *the act of selling* |
| para *money* | satmak *to sell* | serme *the act of spreading* |
| sabah *morning* | sermek *to spread* | tatma *the act of tasting* |
| sigara *cigarette* | tatmak *to taste* | taşma *the act of overflowing* |
| tarak *comb* | taşmak *to overflow* | yakma *the act of burning* |
| toplum *community* | yakmak *to burn* | |
| yatak *bed* | | |

| Nonwords | | | |
|---|---|---|---|
| alıf | gep | apuk | gaj |
| ab | gıcar | aj | genzit |
| aruy | hosu | apran | hesel |
| bıkaf | inser | botkan | ircin |
| banım | kof | berzik | kenyip |
| cava | kitel | cuto | küç |
| çiren | meyu | çifre | merki |
| dopul | pese | deset | pepi |
| eknez | sef | evsol | süp |
| fıran | teley | fazur | tapul |
